# Education Improves Perceived Control but Not Risk Identification in Adolescents Regarding Fentanyl

**DOI:** 10.3390/children12060794

**Published:** 2025-06-17

**Authors:** Christine Bakos-Block, Francine R. Vega, Marylou Cardenas-Turanzas, Bhanumathi Gopal, Tiffany Champagne-Langabeer

**Affiliations:** 1 Center for Behavioral Emergency and Addiction Research, McWilliams School of Biomedical Informatics, The University of Texas Health Science Center at Houston, Houston, TX 77030, USA; christine.bakosblock@uth.tmc.edu (C.B.-B.); maria.cardenasturanzas@uth.tmc.edu (M.C.-T.); bhanumathi.gopal@uth.tmc.edu (B.G.); 2 School of Public Health, University of Texas, Houston, TX 77030, USA; francine.r.vega@uth.tmc.edu

**Keywords:** substance use, adolescent, fentanyl, overdose, education

## Abstract

Background/Objectives: In 2022, 2.2 million adolescents were diagnosed with substance use disorders, including 265,000 with opioid use disorder. The National Survey on Drug Use and Health revealed that 130,000 adolescents misused prescription pain medications, often obtaining them from friends or relatives. This age group perceives weekly heroin use as less risky than those younger or older. Methods: A questionnaire was developed for 7th to 12th graders in a rural Texas school district as part of a fentanyl awareness curriculum. The questionnaire included Likert scale, multiple choice, and yes/no questions. The participants were categorized into younger (grades 7th and 8th) and older students (grades 9th through 12th), and associations were explored between demographic characteristics, responses, and grade groups using chi-square tests. To assess confidence, behavior, and the impact of education, we used chi-square and Fisher’s exact tests. Results: The participants (*n* = 94; 85.11%) identified as Hispanic or Latino, with a smaller percentage identifying as White or more than one race. An association was found between feeling more in control of actions related to substances and fentanyl (*p*-value = 0.04) after receiving education. No association was found between education and confidence in identifying fentanyl. Conclusions: This study aligns with a surge in fentanyl-related overdose deaths in a high-intensity drug trafficking region. Recent fentanyl overdoses among school-age children prompted legislative changes in 2023, making this study valuable for understanding the epidemic within the geographical context. These results suggest that school-based education may play a role in strengthening adolescents’ behavioral intentions to fentanyl exposure, though additional efforts are needed to improve risk identification.

## 1. Introduction

### 1.1. Opioid Trends and Overdose Deaths

Substance use among adolescents aged 12–17 years is a significant public health crisis. In 2022, 2.2 million adolescents were diagnosed with a substance use disorder (SUD). Of those 2.2 million, 265,000 reported having an opioid use disorder [1]. According to the National Survey on Drug Use and Health, 130,000 adolescents reported initiating prescription pain medication misuse in the past year [1]. Nearly forty-five percent (44.9%) reported that they obtained prescription pain relievers from a friend or relative (being given, buying, or stealing). This age cohort is less likely to consider weekly heroin use a significant risk when compared to younger and older adults [1]. Millions of children and adolescents are at risk for severe harm and hospitalization stemming from intentional and unintentional exposure to opioids [2,3,4]. Opioid-related emergency room visits exceeded 59,000 between 2014 and 2017, with 68% of the visits being due to overdoses [2]. Mortality rates associated with SUD have also sharply risen among adolescents in the United States, increasing 94% from 2019 to 2020 and 20% from 2020 to 2021 [5].

### 1.2. Perceptions of Prescription Opioids

The rise in opioid use in adolescents may be partially related to the belief that prescriptions are safe. When surveyed, adolescents believed that prescribed opioids were safe to take, even if they were prescribed to someone else [6]. Youth believed that any medication prescribed by a doctor was safe. Adolescents who receive a legitimate prescription before the 12th grade were 33% more likely to report opioid misuse post-high school [7,8]. Interestingly, even youth who are considered low-risk or those with little to no prior experience with substances who also condemn illicit drug use are at increased risk of misuse when prescribed opioids in high school [7,8,9,10].

### 1.3. Fentanyl in Polydrug Use

The widespread use of illicitly manufactured fentanyl has increased the danger of accidental overdose despite the decline in rates of illicit drug use. Fentanyl exposure is not limited to illicitly manufactured opioids. Illicitly manufactured stimulants that are popular among youth have been found to contain both methamphetamine and trace amounts of fentanyl [11,12,13]. Diversion and non-medical use of prescription stimulants are common in adolescents; approximately one in six adolescents report using prescription stimulants, medically or non-medically [14]. Stimulant misuse increases the incidence of SUD in adulthood [15]. Identifying what adolescents know and understand about opioids and fentanyl risk across ages within this vulnerable population may help in developing effective protective and preventive measures.

### 1.4. Policy

Texas recently passed legislation codifying education surrounding fentanyl in the public school system. House Bill 3908 of the 88th Texas Legislature, Regular Session, 2023, is colloquially known as “Tucker’s Law” [16]. This law requires that public school students receive age-appropriate health education on fentanyl abuse prevention and drug poisoning awareness, declaring October as Fentanyl Poisoning Awareness Month. Prior to the implementation of this law, we sought to evaluate public school students’ current knowledge and beliefs in one rural community in Texas.

### 1.5. Theoretical Framework

The Theory of Planned Behavior (TPB) is a well-established theoretical framework for understanding intentions and behavior [17]. This theory posits that attitudes, subjective norms, and perceived behavioral control collectively influence behavioral intentions, which can predict actual behavior. Previous research supports applying TPB to health behaviors and alcohol use disorder in adults and adolescents. A meta-analysis of TPB found that adults’ attitudes had a stronger relationship to intentions than those of adolescents [18]. Applying TPB, this study aims to offer a nuanced understanding of the factors driving adolescents’ attitudes and intentions toward fentanyl and illicit substance use.

## 2. Materials and Methods

### 2.1. Study Overview

As part of a fentanyl awareness curriculum planning project, in collaboration with a rural Texas school district, a questionnaire was created to better understand adolescents’ knowledge, attitudes, and behaviors surrounding fentanyl in 7th through 12th grade (total enrollment *n* = 4488). The questionnaire was based on the Theory of Planned Behavior (TPB). The Supplement (Appendix A) contains a complete list of questions. The questionnaire contained fourteen Likert scale questions, one multiple-choice question, and three dichotomous yes/no questions. School personnel administered the questionnaire to 100 adolescents on paper, and all responses were anonymous. To ensure the students understood the survey items, school personnel reviewed the questions for age-appropriate readability and comprehension. During administration, staff provided verbal instructions and were available to clarify unfamiliar terms without influencing responses. All students present on the day the survey was administered were invited to participate unless they declined. This study was reviewed by the Institutional Review Board at UTHealth (IRB number HSC-SBMI-24-0528) and was determined to be non-human subjects research under federal guidelines, as it involved secondary analysis of fully deidentified data. All data were collected by school personnel, and no identifiable information was provided to the research team.

### 2.2. Statistical Analysis

We separated the participants according to school grade levels into younger students (grades 7th and 8th) and older students (grades 9th through 12th). We calculated proportions for ordinal and nominal variables and used chi-square tests to assess associations between selected survey responses and grade groups. We tested the null hypothesis that there is no association between the perception of behavior control (e.g., “I feel in control of my actions in situations involving substances and fentanyl”) and whether adolescents reported receiving fentanyl education. To assess this, we used chi-square and Fisher’s exact tests across four key behavior-related questions. These included perceived ability to take precautions, confidence in identifying fentanyl, perceived control in substance-related situations, and intention to take precautions. The analyses were two-tailed, with a significance threshold of *p* < 0.05. All analyses were conducted using Stata I/C version 15 (StataCorp, LLC, College Station, TX, USA).

## 3. Results

### 3.1. Sociodemographic Characteristics of the Sample

A total of 94 of the 100 surveys were used for the analysis. Six surveys were excluded from this study because they did not reach the completion threshold. A majority of survey respondents were male (61.70%, *n* = 58) compared to female (37.23%, *n* = 35), with one student not reporting their gender. The ages of the participants ranged from 12 to 18 years, in grades 7 through 12, with many respondents in the 10th grade (34.04%, *n* = 32). Most of the students (85.11%, *n* = 80) identified as Hispanic or Latino, with 5.32% (*n* = 5) of the students identifying as White and 5.32% (*n* = 5) identifying as more than one race. Table 1 exhibits a summary of the demographic characteristics.

### 3.2. Knowledge and Attitudes Regarding Fentanyl

Nearly 72% (*n* = 67) of the student participants agreed or strongly agreed that they previously heard of fentanyl, with 83.33% (*n* = 75) of the participants indicating “I know what fentanyl is and its potential dangers.” Approximately 87% of the participants (*n* = 79) agreed or strongly agreed with the statement “Fentanyl is highly potent and dangerous,” and 78.02% (*n* = 71) agreed or strongly agreed with the statement “Accidentally taking fentanyl is a serious concern.”

### 3.3. Perceived Risk of Exposure, Norms, and Behavioral Control

Of all participants, 72.22% (*n* = 65) believed that there is a risk of taking fentanyl, even unintentionally, and 76 participants (80.85%) were confident in their ability to take precautions to avoid taking fentanyl accidentally. However, on the questions “accidentally taking fentanyl is something I worry about” and “I am confident in my ability to identify substances that may contain fentanyl” related to the perceived risk of accidental exposure and perceived behavioral control, the agreement was lower at 30.43% and 48.94%, respectively. Fifty-nine students (66.44%) felt strongly that they were in control of their actions in situations involving substances and fentanyl. The details are shown in Table 2.

### 3.4. Intentions and Behavior

A total of 77% (*n* = 68) intended to take precautions to avoid accidentally taking fentanyl. When asked if they had ever been in a situation where they were unsure if a substance contained fentanyl, nearly one-fourth of the participants (24%, *n* = 21) indicated they had. Among those who were not sure, 13% (*n* = 11) mentioned they would still consider using the substance despite not knowing if it contained fentanyl.

### 3.5. Knowledge and Behavior

We examined the association between receiving fentanyl education and adolescents’ perceptions of behavioral control and intentions to avoid fentanyl. No association was found between education and perceived ability to take precautions, confidence in identifying fentanyl, or intention to avoid fentanyl. However, an association was found between the perception of control in substance-related situations and having received fentanyl education (*p* = 0.04), suggesting that students who reported receiving education were more likely to perceive themselves as in control of their actions involving substances and fentanyl (Table 3).

## 4. Discussion

### 4.1. Perceived Risk of Fentanyl Overdose

This research evaluated adolescents’ current knowledge and beliefs about fentanyl in a rural community in Texas. Schools in Texas have experienced a surge of student overdoses, on and off campus, prompting the Texas Legislature to create policies increasing access to overdose-reversal drugs on school campuses and a new law, HB 3908 or Tucker’s Law [16]. Inspired by the overdose death of a 19-year-old who purchased illicit pills containing fentanyl on social media, public schools are required to administer fentanyl prevention and awareness education to students in grades 6 through 12 annually.

Even with a reported decline in illicit drug use among this age group, the prolific use of illicitly manufactured fentanyl in counterfeit pills is credited with the increases in fatal overdoses [19,20]. According to the Centers for Disease Control’s (CDC) State Unintentional Drug Overdose Reporting System (SUDORS), about 90% of all overdose deaths involved opioids, and 83.9% of those involved fentanyl; however, only 35% of cases had any documented history of opioid use [21]. Evidence of counterfeit pills containing illicitly manufactured fentanyl was found in nearly 25% of cases [21]. Misuse of opioids in adolescents acts as a significant precursor to several concerning opioid behaviors, including OUD and opioid overdose [22].

Most students in our survey indicated agreement that fentanyl is dangerous. However, only 28% were worried about accidentally taking fentanyl. Given the association of feeling in control of their actions after being given education about fentanyl, education in the schools may be a protective measure. However, with 52% of the students feeling confident in identifying substances that may contain fentanyl, the result may reflect overconfidence or a gap in accurate risk identification and an area where improvement in education is needed.

### 4.2. Perception of Confidence in the Ability to Control Behavior

A majority of adolescents (76%) in our survey expressed the belief in their ability to take precautions to avoid accidental exposure to fentanyl; however, less than half were confident in their ability to actually identify substances that may contain fentanyl. These findings align with existing literature suggesting that, while adolescents may acknowledge the dangers of fentanyl, their confidence in avoiding contaminated substances is often overestimated. Prior research has shown a gap between risk awareness and actual behavioral capacity, especially in the context of counterfeit pills [7,11,20]. Our study supports the Theory of Planned Behavior’s emphasis on perceived behavioral control as a driver of intention; however, it also adds nuance by highlighting that education may increase confidence in self-control without necessarily improving actual knowledge or skills in substance identification. This echoes the findings in broader adolescent health behavior studies, which suggest that increased confidence does not always equate to safer behavior unless paired with skills-based interventions [17,18].

These findings are consistent with data from *Monitoring the Future*, an annual survey of adolescents conducted by researchers at the University of Michigan. The survey reported that 22.9% of eight graders and 52.9% of 12th graders perceived significant risk associated with taking narcotics. However, risk perception declined when adolescents were asked about prescription stimulants like Adderall, with only 28.1% of eighth graders and 39.6% of 12th graders indicating concern [7]. This disparity mirrors our results, which showed high levels of agreement that fentanyl is dangerous but much lower confidence in adolescents’ ability to identify fentanyl-containing substances. The discrepancy suggests that, while awareness of risk may increase with age or education, gaps in specific substance identification skills persist, highlighting the need for more targeted, skills-based prevention efforts.

This research was conducted in a rural community near Houston, a large metropolitan city in Texas. Houston is the fourth largest city in the United States and serves as a gateway and national distribution hub for illicit substances from the Texas–Mexico border region [23]. Counterfeit prescription drugs, such as hydrocodone, Percocet, and alprazolam, are easily purchased through social media and increasingly contain fentanyl or fentanyl analogs [24]. Counterfeit pills are often indistinguishable from authentically manufactured medications and present a significant risk of fatal overdose to adolescents who engage in experimental substance use [11,25]. Preventative measures, such as educating the youth about fentanyl, represent an effective strategy for equipping adolescents with the skills and awareness needed to navigate high-risk situations involving fentanyl.

## 5. Conclusions

### 5.1. Summary of Findings

This study assessed how fentanyl awareness curriculum influenced adolescents’ knowledge, behavioral control, and intention to avoid exposure to fentanyl. While students generally recognized the dangers of fentanyl, a smaller proportion demonstrated concern about accidental exposure. Notably, education was associated with greater perceived control, although confidence in identifying fentanyl-containing substances remained inconsistent. These findings offer a timely contribution given the increasing prevalence of counterfeit pills and legislative mandates for prevention education in schools.

### 5.2. Limitations and Strengths

Our study findings should be interpreted in light of several limitations. Although surveys were anonymous, this study consisted of self-report data on sensitive topics, where the students may have censored their responses. The sample was non-randomized in one city. Moreover, our study assessed adolescents’ knowledge and behavior surrounding fentanyl. In addition to the limitations stated above, race, ethnicity, and sex were demographically homogenous and may be confounded with social disadvantage. All findings should be placed in context within this limitation.

Despite these limitations, this study contributes valuable insights to adolescent substance use prevention efforts. Our findings suggest that education can bolster adolescents’ sense of agency and perceived behavioral control, which may serve as protective factors against unintentional fentanyl exposure. However, the discrepancy between confidence and actual risk perception underscores the need for educational initiatives that go beyond awareness and emphasize behavioral skills, harm reduction, and critical thinking about substance use. By examining these dynamics in a rural, high-risk region, this research enhances our understanding of how fentanyl prevention programs can be tailored for impact and equity.

## Figures and Tables

**Table 1:** Demographics of survey participants (*n* = 94).

Demographics	*n* = 94 (%)
**Grade**	
7	11 (11.70)
8	10 (10.64)
9	17 (18.09)
10	32 (34.04)
11	17 (18.09)
12	7 (7.45)
**Gender**	
Male	58 (61.70)
Female	35 (37.23)
Prefer not to say	1 (1.06)
**Race/Ethnicity**	
Hispanic or Latino	80 (85.11)
White	5 (5.32)
Multiracial	5 (5.32)
Other	2 (2.13)
Prefer not to say	2 (2.13)

**Table 2:** Comparison of responses to survey questions on knowledge, attitudes, risk, and intentions surrounding fentanyl by student grade level.

	Grades 7–8 *n* = 21 (22.34%)	Grades 9–12 *n* = 73 (77.66%)	Total *n* = 94 (%)	*p*-Value
***Knowledge of fentanyl***				
(2.1) I have heard of fentanyl (*n* = 93)				0.31 *
Strongly disagree/disagree	5 (23.81)	8 (11.11)	13 (13.98)	
Neutral	2 (9.52)	11 (15.28)	13 (13.98)	
Strongly agree/agree	14 (66.67)	53 (73.61)	67 (72.04)	
(2.2) I have heard what fentanyl is and its potential danger (*n* = 90)				0.95 *
Strongly disagree/disagree	2 (10.56)	9 (12.68)	11 (12.22)	
Neutral	1 (5.26)	3 (4.23)	4 (4.44)	
Strongly agree/agree	16 (84.21)	59 (83.10)	75 (83.33)	
Have you received education on fentanyl? (*n* = 88)				0.30 *
Yes	10 (55.56)	48 (68.57)	58 (65.91)	
No	8 (44.44)	22 (31.43)	30 (34.09)	
***Attitudes toward fentanyl***				
(3.1) Fentanyl is highly potent and dangerous (*n* = 91)				0.20 *
Strongly disagree/disagree	3 (15.0)	4 (5.63)	7 (7.69)	
Neutral	0	5 (7.04)	5 (5.49)	
Strongly agree/agree	17 (85.0)	62 (87.32)	79 (86.81)	
(3.2) Accidentally taking fentanyl is a serious concern (*n* = 91)				0.40 *
Strongly disagree/disagree	3 (15.0)	11 (15.49)	14 (15.38)	
Neutral	0	6 (8.45)	6 (6.59)	
Strongly agree/agree	17 (85.0)	54 (76.06)	71 (78.02)	
***Perceived risk of accidental exposure***				
(4.1) I believe there is a risk of taking fentanyl, even if it is not intentional (*n* = 90)				0.69 *
Strongly disagree/disagree	1 (5.0)	8 (11.43)	9 (10.0)	
Neutral	4 (20.0)	12 (17.14)	16 (17.78)	
Strongly agree/agree	15 (75.0)	50 (71.43)	65 (72.22)	
(4.2) Accidentally taking fentanyl is something I worry about (*n* = 92)				0.74 *
Strongly disagree/disagree	9 (45.0)	26 (36.11)	35 (38.04)	
Neutral	6 (30.0)	23 (31.94)	29 (31.52)	
Strongly agree/agree	5 (25.0)	23 (31.94)	28 (30.43)	
***Subjective norms***				
(5.1) People important to me believe I should be cautious about fentanyl (*n* = 93)				0.09 *
Strongly disagree/disagree	6 (28.57)	7 (9.72)	13 (13.98)	
Neutral	3 (14.29)	11 (15.28)	14 (15.05)	
Strongly agree/agree	12 (57.14)	54 (75.0)	66 (70.97)	
(5.2) People whose opinions I value, think that accidentally taking fentanyl is a significant risk (*n* = 94)				0.17 *
Strongly disagree/disagree	3 (14.29)	3 (4.11)	6 (6.38)	
Neutral	2 (9.52)	14 (19.18)	16 (17.02)	
Strongly agree/agree	16 (76.19)	56 (76.71)	72 (76.70)	
***Perceived behavioral control***				
(6.1) I believe I can take precautions to avoid accidentally taking fentanyl (*n* = 94)				0.12
Strongly disagree/disagree	2 (9.52)	4 (5.48)	6 (6.38)	
Neutral	0	12 (16.44)	12 (12.77)	
Strongly agree/agree	19 (9.48)	57 (78.08)	76 (80.85)	
(6.2) I am confident in my ability to identify substances that may contain fentanyl (*n* = 97)				0.94
Strongly disagree/disagree	5 (23.81)	18 (24.66)	23 (24.47)	
Neutral	6 (28.57)	19 (23.06)	25 (26.60)	
Strongly agree/agree	10 (47.62)	36 (49.32)	46 (48.94)	
(6.3) I feel in control of my actions in situations involving substances and fentanyl (*n* = 93)				0.91
Strongly disagree/disagree	4 (19.44)	14 (19.04)	18 (19.35)	
Neutral	3 (14.29)	13 (14.29)	16 (17.20)	
Strongly agree/agree	14 (66.67)	45 (66.67)	59 (66.44)	
***Intentions and behavior***				
(7.1) I intend to take precautions to avoid accidentally taking fentanyl (*n* = 88)				0.83
Strongly disagree/disagree	1 (5.56)	8 (11.43)	9 (10.23)	
Neutral	2 (11.11)	9 (12.86)	11 (12.50)	
Strongly agree/agree	15 (83.33)	53 (75.71)	68 (77.27)	
Have you ever been in a situation where you were unsure if a substance contained fentanyl? (*n* = 87)				0.57
Yes	5 (29.41)	16 (22.86)	21 (24.14)	
No	12 (70.59)	54 (77.14)	66 (75.86)	
If you were unsure if a substance contains fentanyl, would you still consider using it? (*n* = 86)				0.02
Yes	5 (29.41)	6 (8.70)	11 (12.79)	
No	12 (70.59)	63 (91.30)	75 (87.21)	

* *n* = differences were the result of no answer to the question.

**Table 3:** Association of receiving education on fentanyl and the students’ perceived confidence and control.

	No *n* (%)	Yes *n* (%)	*p*-Value
Have you received education on fentanyl? (*n* = 88)	30 (34.09)	58 (65.91)	
(6.1) I believe I can take precautions to avoid accidentally taking fentanyl (*n* = 88)			0.99
Strongly disagree/disagree	2 (6.67)	4 (6.90)	
Neutral	4 (13.33)	8 (13.79)	
Strongly agree/agree	24 (80.00)	46 (79.31)	
(6.2) I am confident in my ability to identify substances that may contain fentanyl (*n* = 88)			0.19
Strongly disagree/disagree	11 (36.67)	11 (18.97)	
Neutral	7 (23.22)	17 (29.31)	
Strongly agree/agree	12 (40.00)	30 (51.72)	
(6.3) I feel in control of my actions in situations involving substances and fentanyl (*n* = 87)			0.04 **
Strongly disagree/disagree	10 (33.33)	8 (14.04)	
Neutral	2 (6.67)	13 (22.81)	
Strongly agree/agree	18 (60.00)	36 (63.16)	
(7.1) I intend to take precautions to avoid accidentally taking fentanyl (*n* = 84)			0.99
Strongly disagree/disagree	3 (10.00)	6 (11.11)	
Neutral	4 (13.33)	7 (12.96)	
Strongly agree/agree	23 (76.67)	41 (75.93)	

** Fisher’s exact test.

## Data Availability

The data that support the findings of this study are not publicly available due to privacy and confidentiality considerations. Interested researchers may contact the corresponding author to discuss potential access, subject to appropriate data use agreements and ethical approvals.

## Abbreviations

The following abbreviations are used in this manuscript:

DOAJ	Directory of open access journals
SUD	Substance use disorder
CDC	Centers for Disease Control
SUDORS	State Unintentional Drug Overdose Reporting System

## Appendix A

### Appendix A.1. Fentanyl Education and Awareness, Questions Based on the Theory of Planned Behavior (Ajzen, 2011)

2.1. I have heard of fentanyl

2.2. I have heard what fentanyl is and its potential dangers

3.1. Fentanyl is highly potent and dangerous

3.2. Accidentally taking fentanyl is a serious concern

4.1. I believe there is a risk of taking fentanyl, even if it’s not intentional

4.2. Accidentally taking fentanyl is something I worry about

5.1. People important to me believe I should be cautious

5.2. People whose opinions I value think that accidentally taking fentanyl is a significant risk

6.1. I believe I have the ability to take precautions to avoid accidentally taking fentanyl

6.2. I am confident in my ability to identify substances that may contain fentanyl

7.1. I intend to take precautions to avoid accidentally taking fentanyl
